# Diagnostic Uncertainty in Dyspneic Patients with Cancer in the Emergency Department

**DOI:** 10.5811/westjem.2020.10.48091

**Published:** 2021-01-29

**Authors:** Katherine M. Hunold, Jeffrey M. Caterino, Jason J. Bischof

**Affiliations:** The Ohio State University Wexner Medical Center, Department of Emergency Medicine, Columbus, Ohio

## Abstract

**Objective:**

Dyspnea is the second most common symptom experienced by the approximately 4.5 million patients with cancer presenting to emergency departments (ED) each year. Distinguishing pneumonia, the most common reason for presentation, from other causes of dyspnea is challenging. This report characterizes the diagnostic uncertainty in patients with dyspnea and pneumonia presenting to an ED by establishing the rates of co-diagnosis, co-treatment, and misdiagnosis.

**Methods:**

Visits by individuals ≥18 years old with cancer who presented with a complaint of dyspnea were identified using the National Hospital Ambulatory Medical Care Survey between 2012–2014 and analyzed for rates of co-diagnosis, co-treatment (treatment or diagnosis for >1 of pneumonia, chronic obstructive pulmonary disease [COPD], and heart failure), and misdiagnosis of pneumonia. Additionally, we assessed rates of diagnostic uncertainty (co-diagnosis, co-treatment, or a lone diagnosis of dyspnea not otherwise specified [NOS]).

**Results:**

Among dyspneic cancer visits (1,593,930), 15.2% (95% confidence interval [CI], 11.1–20.5%) were diagnosed with pneumonia, 22.5% (95% CI, 16.7–29.7%) with COPD, and 7.4% (95% CI 4.7–11.4%) with heart failure. Dyspnea NOS was diagnosed in 32.3% (95% CI, 25.7–39.7%) of visits and as the only diagnosis in 23.1% (95% CI, 16.3–31.6%) of all visits. Co-diagnosis occurred in 4.0% (95% CI, 2.0–7.6%) of dyspneic adults with cancer and co-treatment in 12.1% (95% CI, 7.5–18.9%). Agreement between emergency physician and inpatient documentation for presence of pneumonia was 57.7% (95% CI, 37.0–76.1%).

**Conclusion:**

Diagnostic uncertainty remains a significant concern in patients with cancer presenting to the ED with dyspnea. Clinical uncertainty among dyspneic patients results in both misdiagnosis and under-treatment of patients with pneumonia and cancer.

## INTRODUCTION

Diagnosing the etiology of shortness of breath or dyspnea in emergency department (ED) patients is challenging. ED providers frequently co-diagnose and co-treat multiple pathologies simultaneously, particularly pneumonia, heart failure (HF), and chronic obstructive pulmonary disease (COPD).^1^ Diagnostic uncertainty, defined as either co-treatment or co-diagnosis, is compounded in patients with cancer due to multiple patient- and disease-specific factors. The immune response in patients with cancer may be altered due to immunosuppression, obscuring key symptoms that aid in diagnosis.^2^–^4^ Additionally, the presence of effusions and malignant infiltrates can confound imaging results.^5^, ^6^ Diagnostic uncertainty is particularly concerning as it negatively impacts multiple, patient-centered outcomes including increased rates of unnecessary admission, longer lengths of stay, and increased mortality.^7^–^9^ Investigating the diagnostic accuracy associated with dyspnea in this special population is warranted, particularly as there are an estimated 4.5 million yearly visits to EDs by patients with cancer in the United States.^10^, ^11^ Among this population the symptom of dyspnea is the second most common reason for presentation to the ED.^12^

The appropriate diagnosis and treatment of infectious processes is of particular importance in a cancer patient with a compromised immune system. In particular, pneumonia is a common known complication of systemic therapy and radiotherapy and has been strongly associated with admission and mortality.^13^ Retrospective data reveals pneumonia is the most common ED diagnosis for cancer-related visits (4.5%, or approximately 200,000 annual visits) and is associated with a high rate of admission (89%).^10^ Appropriate identification of infectious pneumonia predicates appropriate treatment initiation, risk stratification, and disposition.

We examined a sample of patients with cancer presenting to the ED for acute care using a national database. The objective was to identify the rates of co-diagnosis, co-treatment and diagnostic uncertainty among common causes of dyspnea in this sample. We also sought to identify the proportion of patients diagnosed with pneumonia in this sample and the degree of misdiagnosis by emergency physicians by assessing the level of agreement between emergency physicians and inpatient physicians for the diagnosis of pneumonia.

## METHODS

### Study Setting and Population

The National Hospital Ambulatory Medical Care Survey (NHAMCS) is conducted annually to describe ambulatory emergency care at US hospitals.^14^ We included data from calendar years 2012 (when the cancer variable was introduced) to 2014. Data from 2015 and beyond were excluded due to the conversion of the *International Classification of Diseases* (ICD) categorization during the 2015 calendar year, limiting direct comparison to prior literature. We identified visits by individuals aged ≥18 years old with a history of cancer presenting with a complaint of dyspnea using the NHAMCS cancer variable. The following reason for visit codes for dyspnea were used: 1415.0 (shortness of breath); 1420.0 (labored or difficult breathing [dyspnea]); 1425.0 (wheezing); 1430.0 (breathing problems); 1430.1 (disorders of respiratory sound); and 1403.2 (rapid breathing).^1^

To allow comparison with previous literature^1^, ^15^ and to exclude patients who had clear etiologies of their dyspnea (eg, atrial fibrillation with rapid ventricular response), we limited analyses of co-treatment, co-diagnosis, and diagnostic uncertainty to the subset of patients with ED diagnoses of pneumonia, COPD, or HF.

### Key Outcome Measures and Definitions

The primary outcomes were the proportion of ED visits with pneumonia diagnosis, co-diagnosis (>1 diagnosis of pneumonia, COPD, and HF), and co-treatment (treatment for >1 etiology). We included treatment in addition to diagnosis, as ED documentation of diagnoses is known to be incomplete and may not accurately represent whether the treating physician felt a condition was present. For admitted patients, we compared the agreement of ED pneumonia, COPD, and HF diagnosis with hospital discharge diagnosis.

**Population Health Research Capsule**

What do we already know about this issue?

*Dyspnea is the second most common reason for presentation to the ED by patients with cancer. Distinguishing pneumonia from other causes of dyspnea in this population is challenging*.

What was the research question?

*We sought to characterizes the diagnostic uncertainty in patients with cancer presenting to an ED with dyspnea and pneumonia*.

What was the major finding of the study?

*Diagnostic uncertainty in ED patients with cancer and dyspnea results in both misdiagnosis and under-treatment of pneumonia*.

How does this improve population health?

*Improved ED diagnostic accuracy in patients with cancer and dyspnea could improve morbidity and mortality*.

ED and hospital diagnoses of pneumonia were defined as ICD-9-CM codes 480.xx, 481, 482.0, 482.1, 482.2, 482.30, 482.31, 482.32, 482.39, 482.4x, 482.8x, 482.9, 483.xx, 485, 486, 487.0 and 488.11; COPD as codes 491.21, 491.22, 491.8, 491.9, 492.8, 493.2xx and 496; and HF as codes 402.01, 402.11, 402.91, 404.01, 404.03, 404.11, 404.13, 404.91, and 428.xx. Dyspnea not otherwise specified (NOS) was defined as ICD-9-CM code 786.^1^, ^15^

Treatment for pneumonia, COPD, and HF were determined based on ED medications administered that were distinct for one of these conditions using the drug categories in NHAMCS and concordant with work by our group and others.^1^, ^15^ Pneumonia treatment included penicillin, cephalosporin, fluoroquinolone, macrolide, vancomycin, tetracycline, aminoglycoside, or carbapenem antibiotics. Treatment for COPD included glucocorticoids. Treatment for HF included loop diuretics, vasodilators, or positive inotropes.^1^, ^14^, ^15^ Inpatient diagnosis of pneumonia was used as the criterion standard to determine the rate of misdiagnosis by ED providers given the lack of culture and imaging results in the dataset.

### Data Analysis

Descriptive statistics are reported. Confidence intervals and *P*-values are not reported, as statistical significance would not correlate with clinical significance given large weighted sample sizes in the dataset. We used NHAMCS weighting procedures as outlined in their documentation to obtain nationally representative estimates.^14^ For strata with a single sampling unit, standard deviations calculated using both centered and certainty in STATA had similar results. We incorporated appropriate elements from published recommendations for NHAMCS analyses.^16^ Data management was conducted using SAS 9.4 (SAS Institute, Inc., Cary, NC) and data analysis using STATA 15 (StataCorp., College Station, TX).

This study was determined to be exempt from institutional review board review.

## RESULTS

From 2012–2014, the NHAMCS contained 2464 visits representing 1,593,930 weighted ED visits by dyspneic adults with cancer. This population is described in Table 1 overall and stratified by disposition. Multiple etiologies of dyspnea exist. Table 2 reports the ED diagnosis and treatment frequency in this patient sample. Of all dyspneic cancer visits in the ED, 15.2% (95% CI, 11.1–20.5%) were diagnosed with pneumonia, 22.5% (95% CI, 16.7–29.7%) with COPD, and 7.4% (95% CI, 4.7–11.4%) with HF. Dyspnea NOS was diagnosed in 32.3% (95% CI, 25.7–39.7%) of visits and was the only diagnosis in 23.1% (95% CI, 16.3–31.6%). Co-diagnosis occurred in 4.0% (95% CI, 2.0–7.6%) of dyspneic adults with cancer and co-treatment in 12.1% (95% CI, 7.5–18.9%). Co-diagnosis of pneumonia with either COPD or HF was present in 2.6% (95% CI, 1.1–6.1%). We did not separately report co-diagnosis of all three diagnoses – pneumonia, COPD, and HF –due to too small sample size per NHAMCS guidelines.

Only 65.6% of all adult visits diagnosed with pneumonia received treatment with one of the antibiotics noted above; 61.2% of COPD visits received treatment; and 66.0% of HF visits received treatment. Imaging utilization was similar in the pneumonia subpopulation (radiograph: 88.8% [n = 215,131], chest computed tomography (CT): 15.4% [n = 37,261]) as in the total dyspneic population (radiograph: 79.3% [1,263,448], chest CT: 15.8% [n = 251,220]). Among hospitalized patients, hospital diagnosis agreement with ED diagnosis of pneumonia, COPD, and HF was low (Table 3). In admitted patients with an ED diagnosis of pneumonia, only 57.7% had a hospital discharge diagnosis of pneumonia. Rates were 45.9% for COPD and 50.3% for HF. In patients with an inpatient diagnosis of pneumonia, 74.6% had an ED diagnosis of pneumonia. Rates were 70.7% for COPD and 65.1% for HF. Among those admitted to the hospital, 168,717 (21.3%) had a length of stay of two days or less. Among those with pneumonia admitted to the hospital, 36,482 (17.9%) had a length of stay of two days or less.

## DISCUSSION

Differentiating the etiologies of dyspnea is challenging and clinically critically important as ED diagnosis is known to affect the subsequent care of patients.^1^, ^17^ Inappropriate treatment of dyspnea secondary to diagnostic uncertainty can result in multiple adverse patient outcomes. The diagnostic uncertainty is further complicated in this population by the natural history of cancer and the potential effects of cancer treatment. This is a significant issue in this population as the proportion with “Dyspnea NOS” as the only diagnosis listed was 23.1%. The rate of co-treatment (12.1%) when compared to co-diagnosis (4.0%) further demonstrates the challenge of diagnostic uncertainty in this population. This may suggest that providers may be ordering additional unnecessary treatment or not listing all relevant diagnoses when faced with diagnostic uncertainty. Alternatively, this may represent a choice to pick a general rather than specific code. Finally, these markers of uncertainty were higher in those admitted compared to discharged; this could reflect that the admitted patient population was more medically complex and/or more ill compared to those who were discharged and confound the results. The rates of co-diagnosis (6%), co-treatment (15%) and the proportion with “Dyspnea NOS” as the only diagnosis (23%) are similar to a population of all dyspneic, older adult ED patients.^1^

Pneumonia diagnosis among patients with cancer presenting to an ED for acute care is common.^10^ In this population, pneumonia was the most common specific diagnosis (13.9%) and was commonly present in those admitted (23.1%). Our analysis reveals a concern for a high rate of pneumonia misdiagnosis and under-treatment. Among individuals hospitalized with pneumonia, only 57.7% were discharged with a diagnosis of pneumonia, suggesting a high rate of over-diagnosis of pneumonia similar to other high-risk subpopulations in the ED setting.^1^ This proportion is lower than previously reported rates for agreement between ED and inpatient diagnosis for community-acquired pneumonia in the US (66.9%, 72%)^18^, ^19^ but higher than a study performed in Israel (29%).^20^ Additionally, among individuals diagnosed with pneumonia only 65.6% were treated with antibiotics, suggesting a high rate of under-treatment in this population. These findings are concerning as it has been demonstrated that inappropriate treatment of dyspnea in the ED and inappropriate treatment of infection in patients is associated with increased mortality.^21^–^23^

Using the inpatient diagnosis of pneumonia as a criterion standard, 25.4% of patients with dyspnea diagnosed as having pneumonia by the inpatient team were not identified by the ED. This is an alarmingly high rate of under-diagnosis and is increased when compared to the 20.4% reported for community-acquired pneumonia in a general ED patient population.^27^ This finding may be attributed to the increased burden of comorbidities and malignancy-related changes (tumor burden, malignant effusions, treatment-related effect) in our cohort. Additionally, this proportion likely represents an overestimate of the problem in this population as a portion of patients likely developed pneumonia during their hospitalization. Under-diagnosis leads to delayed antibiotic initiation, resulting in increased mortality and morbidity. The rate noted in this study requires further investigation to determine the true rates of ED under-diagnosis of pneumonia.

Among those individuals admitted, a fifth experienced a length of stay of two days or less further emphasizing the concern that the initial ED decision to admit a patient with cancer and dyspnea could be modified in a significant number of patients. The high rate of short hospitalization suggests that improved diagnostics or care pathways may be beneficial to improving the care of these patients. This could lead to more appropriate management and disposition decisions for dyspneic patients with cancer, particularly given the high rates of admission once pneumonia is diagnosed.

One potential modality to increase diagnostic accuracy in the ED is CT imaging.^24^, ^25^ In our study, only 15.8% of patients had a CT performed. A study of inpatients with pneumonia in a time period overlapping with our data set found a CT utilization rate of 33%.^26^ It is not surprising there are higher rates of utilization in inpatients as this is likely a sicker population. Additional work would be needed to validate an early-CT strategy in the ED.

## LIMITATIONS

Due to the retrospective nature of this study and the limitations associated with the dataset,^16^, ^28^, ^29^ further characterization of diagnostic uncertainty in the ED of dyspneic patients is not possible. The uncertainty is due to multiple reasons; a prospective study would be required to further assess the outcomes and causes of dyspneic ED patients with cancer. The criterion standard for pneumonia was used as an inpatient diagnosis, but there is no ability to verify the accuracy of this diagnosis. Since we do not know whether inpatient physicians might be under-diagnosing, over-diagnosing, or both we cannot determine which direction bias arising from this problem would move our results. Further, an inpatient discharge diagnosis could reflect a problem that arose while the patient was hospitalized and thus not represent a missed diagnosis by the emergency physician. Future efforts should focus on identifying new diagnostic approaches such as biomarkers or risk stratification algorithms to improve the clinical outcomes of this patient population.

## CONCLUSION

Among patients with cancer presenting to the ED with dyspnea, diagnostic uncertainty remains a significant concern. Clinical uncertainty among dyspneic ED patients results in both misdiagnosis and under-treatment of patients with pneumonia and cancer in the ED setting. There is only moderate agreement between ED and inpatient diagnosis of pneumonia in this population. These results demonstrate a need for further research to accurately diagnose the etiologies of dyspnea in patients with cancer seeking acute care in the ED setting.

## Figures and Tables

**Table 1:** Weighted characteristics of adult dyspneic cancer patient visits by emergency department disposition in calendar years 2012–2014. Data presented as n rounded to nearest 1,000 (%).

	All (n=1,594,000)	Admitted (n=794,000)	Not Admitted (n=800,000)
Age, mean (SD)	69.6 (1.0)	77.2 (1.0)	53.7 (0.8)
Female	711,000 (44.6)	353,000 (44.4)	359,000 (44.8)
Race			
White	994,000 (62.4)	541,000 (68.2)	453,000 (56.6)
Black	117,000 (7.3)	79,000 (9.9)	38,000 (4.7)
Other	62,000 (3.9)	50,000 (6.4)	12,000 (1.5)
Missing	421,000 (26.4)	123,000 (15.5)	298,000 (37.2)
Comorbidities			
COPD	583,000 (36.6)	299,000 (37.7)	284,000 (35.4)
HF	318,000 (19.9)	217,000 (27.4)	101,000 (12.6)
Diabetes	419,000 (26.3)	269,000 (33.8)	150,000 (18.8)
Renal disease*	112,000 (7.0)	95,000 (11.9)	17,000 (2.2)
Residence			
Private residence	1,466,000 (91.9)	706,000 (89.0)	759,000 (94.9)
Nursing home	67,000 (4.2)	56,000 (7.1)	11,000 (1.4)
Other/missing/unknown	61,000 (3.8)	31,000 (3.9)	30,000 (3.8)
Arrived by ambulance	599,000 (37.6)	352,000 (44.4)	247,000 (30.8)

* Variable “EDDIAL” for calendar years 2012–2013; “chronic kidney disease” and “end-stage renal disease” for 2014.

*EDDIAL*, a condition requiring dialysis; *SD*, standard deviation; *COPD*, chronic obstructive pulmonary disease, *HF*, heart failure.

**Table 2:** Weighted diagnosis, co-diagnosis and co-treatment of adult dyspneic cancer patient visits by emergency department disposition in calendar years 2012–2014. Data presented as n rounded to the nearest 1,000 (%).

	All (n=1,594,000)	Admitted (n=794,000)	Not Admitted (n=800,000)	Admission Rate
Top 10 ICD-9 categories diagnosis*				
Symptoms involving respiratory system and other chest symptoms	515,000 (32.3)	206,000 (26.0)	309,000 (38.6)	(40.0)
Pneumonia, organism unspecified	222,000 (13.9)	183,000 (23.1)	39,000 (4.8)	(82.5)
Chronic bronchitis	201,000 (12.6)	100,000 (12.6)	101,000 (12.6)	(49.8)
Chronic airway obstruction	153,000 (9.6)	51,000 (6.4)	102,000 (12.7)	(33.4)
Heart failure (HF)	118,000 (7.4)	74,000 (9.4)	43,000 (5.4)	(63.2)
Cardiac dysrhythmias	116,000 (7.3)	38,000 (4.8)	78,000 (9.7)	(32.8)
Disorders of fluid, electrolyte and acid-base balance	115,000 (7.2)	83,000 (10.5)	32,000 (4.0)	(72.0)
Pleurisy	115,000 (7.2)	82,000 (10.3)	33,000 (4.2)	(71.2)
General symptoms	100,000 (6.3)	50,000 (6.3)	49,000 (6.2)	(50.5)
Malignant neoplasm of trachea, bronchus, lung	90,000 (5.7)	29,000 (3.6)	62,000 (7.7)	(31.5)
Diagnosed with:				
Pneumonia, all types	242,000 (15.2)	203,000 (25.6)	39,000 (4.8)	(84.0)
COPD	359,000 (22.5)	151,000 (19.0)	208,000 (26.0)	(42.0)
HF	118,000 (7.4)	74,000 (9.4)	43,000 (5.4)	(63.2)
≥ 1 of pneumonia, COPD, HF	654,000 (41.1)	382,000 (48.1)	273,000 (34.1)	(58.3)
Pneumonia and COPD or HF	42,000 (2.6)	37,000 (4.7)	5,000 (0.6)	(88.8)
Pneumonia and COPD	39,000 (2.4)	36,000 (4.5)	3,000 (0.4)	(91.6)
Pneumonia and HF	2,000 (0.1)	2,000 (0.2)	0 (0.0)	(100.0)
Dyspnea NOS	515,000 (32.3)	206,000 (26.0)	309,000 (38.6)	(40.0)
Only dyspnea NOS	368,000 (23.1)	111,000 (14.0)	257,000 (32.1)	(30.2)
Co-diagnosis	63,000 (4.0)	47,000 (5.9)	16,000 (2.0)	(74.6)
Co-treatment	193,000 (12.1)	165,000 (20.8)	28,000 (3.5)	(85.4)

* First 3 numerals of ICD-9 diagnosis code as recorded in NHAMCS variables DIAG1–DIAG3

*ICD*, International Classification of Diseases; *COPD*, chronic obstructive pulmonary disease; *NOS*, not otherwise specified.

**Table 3a:** Diagnosis of pneumonia by emergency physician and inpatient providers in dyspneic cancer patients admitted to the hospital (n = 794,000). Data presented as n rounded to the nearest 1,000 (%).

	Hospital Diagnosis	

ED diagnosis	Present	Not present
Present	117,000 (14.8)	86,000 (10.8)
Not Present	40,000 (5.1)	550,000 (69.3)

*ED*, emergency department.

**Table 3b:** Diagnosis of chronic obstructive pulmonary disease (COPD) by emergency physician and inpatient providers in dyspneic cancer patients admitted to the hospital (n = 794,000). Data presented as n (%).

	Hospital Diagnosis	

ED diagnosis	Present	Not present
Present	69,000 (8.7)	82,000 (10.3)
Not Present	29,000 (3.6)	614,000 (77.4)

*ED*, emergency department.

**Table 3c:** Diagnosis of heart failure (HF) by emergency physician and inpatient providers in dyspneic cancer patients admitted to the hospital (n = 794,000). Data presented as n (%).

	Hospital Diagnosis	

ED diagnosis	Present	Not present
Present	37,000 (4.7)	37,000 (4.7)
Not Present	20,000 (2.5)	700,000 (88.1)

*ED*, emergency department.
